# Optimization of Magnetic and Paper-Based Molecularly Imprinted Polymers for Selective Extraction of Charantin in *Momordica charantia*

**DOI:** 10.3390/ijms24097870

**Published:** 2023-04-26

**Authors:** Nantana Nuchtavorn, Jiraporn Leanpolchareanchai, Satsawat Visansirikul, Somnuk Bunsupa

**Affiliations:** 1 Department of Pharmaceutical Chemistry, Faculty of Pharmacy, Mahidol University, 447 Sri-Ayudhaya Rd., Rajathevee, Bangkok 10400, Thailand; satsawat.vis@mahidol.edu; 2 Department of Pharmacy, Faculty of Pharmacy, Mahidol University, 447 Sri-Ayudhaya Rd., Rajathevee, Bangkok 10400, Thailand; jiraporn.lea@mahidol.edu; 3 Department of Pharmacognosy, Faculty of Pharmacy, Mahidol University, 447 Sri-Ayudhaya Rd., Rajathevee, Bangkok 10400, Thailand; somnuk.bun@mahidol.edu

**Keywords:** charantin, magnetic nanoparticles, *M. charantia*, molecularly imprinted polymers, paper-based devices

## Abstract

Charantin is a mixture of β-sitosterol and stigmastadienol glucosides, which effectively lowers high blood glucose. Novel molecularly imprinted polymers coated magnetic nanoparticles (Fe_3_O_4_@MIPs) and filter paper (paper@MIPs) were synthesized by sol-gel polymerization to selectively extract charantin. β-sitosterol glucoside was selected as a template for imprinting a specific recognition owing to its larger molecular surface area than that of 5,25-stigmastadienol glucoside. Factorial designs were used to examine the effects of the types of porogenic solvents and cross-linkers on the extraction efficiency and imprinting factor before investigating other factors (for example, amounts of template and coated MIPs, and types of substrates for MIP immobilization). Compared to traditional liquid–liquid extraction, the optimal Fe_3_O_4_@MIP-based dispersive micro-solid phase extraction and paper@MIP extraction provided excellent extraction efficiency (87.5 ± 2.1% and 85.0 ± 2.9%, respectively) and selectivity. Charantin was well separated, and a new unidentified sterol glucoside was observed using the developed high-performance liquid chromatography with diode-array detection (Rs ≥ 2.0, *n* > 16,400). The developed methods were successfully utilized to extract and quantify charantin from *M. charantia* fruit powder and herbal products. Moreover, these methods are rapid (<10 min), inexpensive, simple, reproducible, and environmentally friendly.

## 1. Introduction

*Momordica charantia* L. (bitter gourd) is a plant-based food belonging to the Cucurbitaceae family, which contains versatile bioactive compounds such as cucurbitane-type triterpenoids and is widely cultivated in tropical and subtropical regions. *M. charantia* was commonly used as a traditional medicine for the treatment of laxative, anthelmintic, and stomachic issues. Notably, *M. charantia* is known for positive effects on lowering blood glucose for the treatment of diabetes and its complications. Additionally, various biological activities of *M. charantia* were reported such as antihyperglycemic, antibacterial, antiviral, antitumor, immunomodulatory, antioxidant, antimutagenic, antiulcer, antifertility, hepatoprotective, and anti-inflammatory activities [1,2].

**Figure 1:**
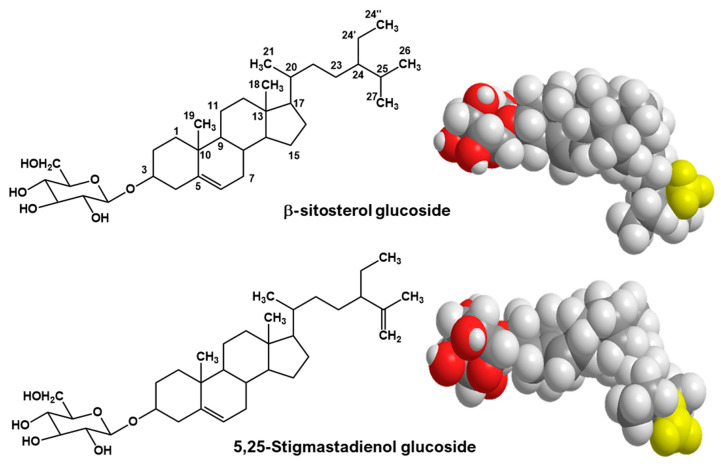
Two- and three-dimensional structures of β-sitosterol glucoside and 5,25-stigmastedienol glucoside.

Among the bioactive moieties, the beneficial effect on diabetes is attributed to steroidal saponins called charantin, which is in higher quantities in fruits [3]. Charantin (Figure 1) was first reported in 1966 as a mixture of the two phytosterol glycosides; β-sitosterol 3-O-β-D-glucoside and 5,22-stigmasterol 3-O-β-D-glucoside [4,5]. However, the structure of charantin given in some publications, as well as by chemical suppliers, is composed of different molecules (a mixture of β-sitosterol 3-O-β-D-glucoside and 5,25-stigmasterol 3-O-β-D-glucoside or 5,25-stigmastadienol glucoside). Moreover, details regarding the identification of this compound have not yet been reported [6].

Several analytical methods were utilized for the quality control of *M. charantia* fruits by determining various active constituents such as five cucurbitane-type triterpenoids in the fruit by reversed-phase high-performance liquid chromatography (HPLC) with evaporative light scattering detection (HPLC-ELSD) [7], and five momordicine and momordicoside derivatives in dietary supplements by HPLC-electrospray ionization—tandem mass spectrometry (MS/MS) [8]. Moreover, various chromatographic techniques, such as high-performance thin layer chromatography (HPTLC) densitometry [9] and HPLC-diode array detection (DAD) [3,10,11], were reported for the quantitation of charantin. These analytical methods are mainly combined with solvent extraction (e.g., ethanol, methanol, hexane) through ultrasonic-assisted liquid–liquid extraction (LLE) [3,11,12], Soxhlet and pressurized extraction [10], and supercritical carbon dioxide extraction [11]. However, these methods involve multi-step extractions and are time-consuming (40 min to 6.5 h), possibly leading to sample loss.

Molecularly imprinted polymers (MIPs) are synthesized using molecular imprinting technology, in which functional monomers are allowed to self-assemble around a template molecule and are subsequently polymerized in the presence of a crosslinker. After completion of polymerization, the templates are removed, providing tailor-made binding sites or artificial receptors that have complementary shapes, sizes, and functionalities toward the target template [13]. Compared to traditional sorbents, MIPs offer good mechanical/chemical stability, high specificity, low-cost, easy preparation, and reversible adsorption/release of the target molecule, enabling selective sample extraction and preconcentration. A combination of MIPs with additional magnetic properties can be prepared by fabricating MIP on the surface of a magnetic substrate such as ferric oxide (Fe_3_O_4_). Fe_3_O_4_@MIPs received considerable attention as dispersive micro-solid-phase extraction (D-µSPE) sorbent. The Fe_3_O_4_@MIPs are simply dispersed in a sample containing the target analyte and stirred to allow the analyte to adsorb onto the magnetic polymers. Fe_3_O_4_@MIPs can then be easily isolated from the samples using an external magnetic separator without filtration or centrifugation. This technique is relatively simple and rapid [14,15].

Similarly, cellulosic paper was used as a porous material platform for sensing devices [16,17], enzymatic reactors [18,19], and MIPs extraction sorbents [20,21]. Paper is a ubiquitous, inexpensive, and biodegradable material that is compatible with a wide range of chemicals. In addition, the paper surface functionalization/modification can be easily employed to alter its properties. Owing to these advantages, paper@MIPs are another promising extraction sorbent for achieving high adsorption capacity and binding specificity. In addition, paper@MIPs can be used as a syringe filter by placing them on the holder connected to the syringe, thus offering fast and simple extraction.

The optimization of extraction methods by investigating various factors influencing extraction efficiency can be time-consuming and laborious; therefore, the statistical design of experiments is an efficient procedure to obtain the ultimate responses. Factorial design is the most commonly used screening design for extraction methods and analytical method development, particularly improving extraction efficiency and chromatographic separation. The evaluation of main variables contributing to the measured responses is the most effective procedure. Moreover, the factorial design study substantially reduces the bias, resources, time, and effort required to solve problems. Thus, factorial design studies are information-rich techniques capable of analyzing analytical data obtaining valid results [22].

**Figure 2:**
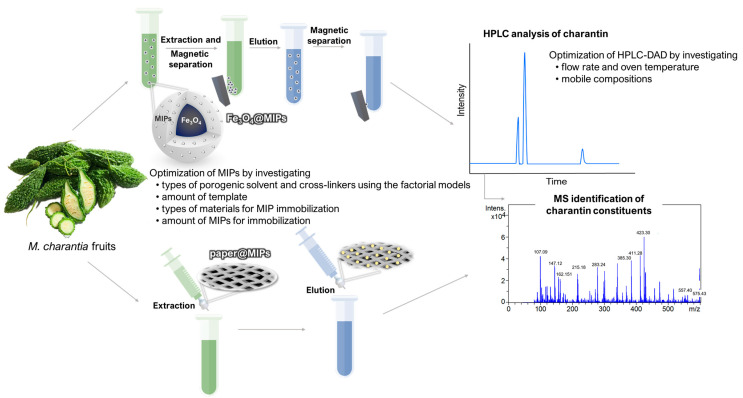
The overall method for MIPs optimization coupled with HPLC analysis of charantin in herbal products.

Herein, the novel Fe_3_O_4_@MIPs-based D-µSPE and paper@MIPs coupled with HPLC-DAD were developed for the rapid and selective extraction and determination of charantin in herbal samples (Figure 2). Although applications of Fe_3_O_4_@MIPs for herbal and food samples were illustrated previously [23,24,25], to the best of our knowledge, studies on the MIP extraction of charantin are yet to be reported. The chromatographic separation of charantin was optimized and validated using HPLC-DAD, following which MIP optimization was performed using a factorial experimental design. The morphology and characterization of the Fe_3_O_4_@MIPs and paper@MIPs surfaces were determined using scanning electron microscopy (SEM) and Fourier transform infrared (FTIR) spectroscopy, respectively. Both Fe_3_O_4_@MIPs and paper@MIPs possess specific recognition sites for charantin binding. Compared to traditional LLE, the method provides excellent extraction efficiency and selectivity, as well as more convenience by minimizing the steps involved in sample preparation, consequently reducing the sample loss associated with the procedure. Finally, the method was successfully applied to analyze charantin in various herbal samples without the purification steps such as preparative HPLC [5].

## 2. Results and Discussion

### 2.1. HPLC-DAD Optimization

**Figure 3:**
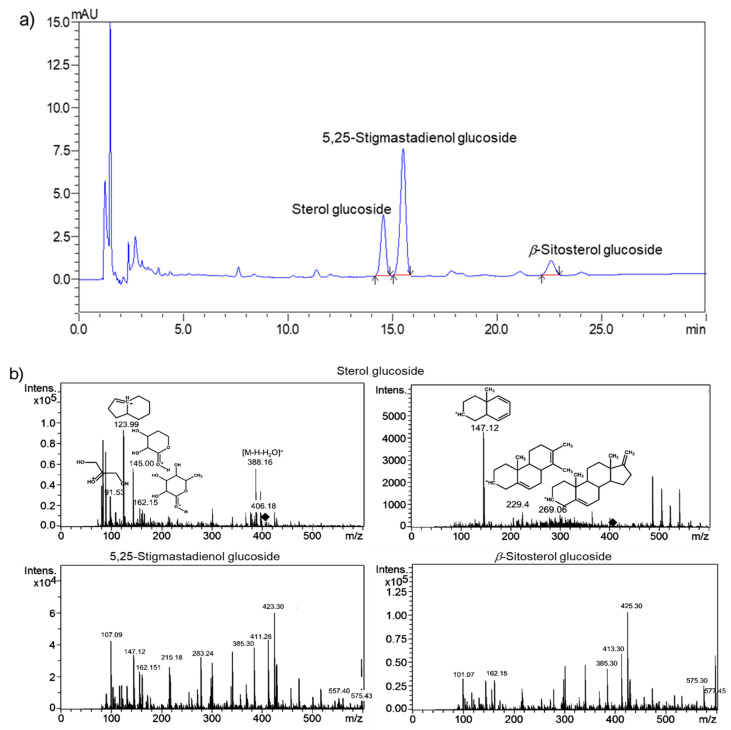
(**a**) Chromatographic separation of charantin under optimal conditions: C18 column (length: 150 mm; diameter: 4.0 mm; particle size: 3 μm), injection volume of 20 μL, isocratic elution using ACN/water (80:20, *v*/*v*), flow rate 0.8 mL/min, oven temperature of 40 °C and detection wavelength of 204 nm and (**b**) mass spectra of charantin constituents.

Desai et al. [5] reported full validated chromatographic conditions for the analysis of charantin, which ensured method transfer for further optimization. However, tailing peaks of charantin were observed and the separation efficiency needed improvement. The initial chromatographic separation condition for charantin was modified by changing column dimension from C18 column 75 mm × 4.6 mm × 3.5 µm to 100 mm × 2.1 mm × 2.6 μm. This modification resulted in a total run time of 11 min with resolution (R_s_) ≥ 2.0. However, tailing and fronting peak shapes (tailing factor (TF) = 1.2 and 0.8) were observed for the 5,25-stigmastadienol and β-sitosterol glucosides, respectively. In addition, a small peak of an unidentified compound co-eluted with 5,25-stigmastadienol glucoside. Therefore, the physical parameters (i.e., the flow rate and oven temperature) were investigated. Changing the flow rate from 0.5 mL/min to 0.6 mL/min did not improve the separation of charantin. Although increasing the oven temperature (25–50 °C) decreased the retention time, 5,25-stigmastadienol glucoside and another small peak were not well separated (Rs < 2.0) at 45 and 50 °C (Figure S1). Therefore, 40 °C was selected as the optimal temperature (Rs ≥ 2.2, *n* > 5000, TF = 0.9–1.4) for further optimization. The mobile phase compositions (methanol (MeOH): water) were evaluated from 98:2 to 95:5 to improve the resolving power and separation efficiency of 5,25-stigmastadienol glucoside, β-sitosterol glucoside, and unidentified compounds. However, the baseline separation of all the peaks could not be achieved (R_s_ < 2.0, *n* < 3500, TF = 0.8–1.6). The decrease in MeOH content lowered the elution strength, resulting in a poor peak shape. Furthermore, changes in the mobile phase composition from MeOH/water to acetonitrile (ACN): water were examined from 85:15 to 80:20. The ACN system yielded a better peak shape than the MeOH system. A ratio of 80:20 offered R_s_ ≥ 2.0, *n* > 16,000, and TF = 0.9–1.0 with a total run time of 30 min, whereas other ratios gave R_s_ < 2.0. In addition, a flow rate in the range of 0.6–1.0 mL/min reduced the run time, a flow rate of 0.8 mL/min provided a run time of <23 min with good analytical performance (R_s_ ≥ 2.0, *n* > 16,000, and TF = 0.9–1.0). The optimal conditions were achieved on a C18 column (length: 150 mm; diameter: 4.0 mm; particle size 3 μm), an injection volume of 20 μL, isocratic elution using ACN/water (80:20, *v*/*v*), a flow rate 0.8 mL/min, an oven temperature of 40 °C, and a detection wavelength of 204 nm (Figure 3a, and Table S1). These conditions offered better peak shapes and separation efficiency than the previous study [5].

Unlike previous studies [5,10], three major peaks of charantin were observed under optimized HPLC-DAD conditions. Therefore, LC-MS/MS analysis of charantin was performed to characterize its components. The spiked β-sitosterol glucoside in the charantin solution confirmed that the last eluted peak was β-sitosterol glucoside, and the presence of the [M + H]^+^ peak at *m/z* 577.30 confirmed the molecular weight (MW) of β-sitosterol glucoside at 576.8 g/mole. From the HPLC studies, the second peak could potentially be 5,25-stigmastadienol glucoside (MW = 574.8 g/mole) owing to the polarity arising from its double bond and the presence of [M + H]^+^ peak of the aglycone fragment at *m/z* 411.29. Finally, the first peak corresponded to the third component of charantin with the highest polarity, which was not reported in the literature. This peak was further analyzed, which revealed an aglycone fragment at *m/z* 406.18 [26,27,28] (Figure 3b). However, further structural identification was required.

### 2.2. Method Validation

A specificity test was performed based on the purity angles of the major peaks that were less than the threshold value. The purity curve was below the threshold across the entire peak, indicating spectral homogeneity across the peak. The linearity, accuracy, precision, and limit of quantitation (LOQ) of the optimized method were also evaluated (Table S2). The responses were linear with *r* = 0.9979–0.9994 in the range of 1–50 µg/mL for charantin and its components. Repeatability and reproducibility showed percent relative standard deviations (%RSDs) of 0.8–3.5% and 1.1–3.9%, respectively. The LOQs and LODs were 0.5–1.0 µg/mL (%RSD ≤ 3.1%) and 0.17–0.34 µg/mL, respectively, demonstrating higher sensitivity than the previous report [5]. The mean recoveries were in the range of 96.9–106.1% with %RSD ≤ 3.4. The recovery data indicated that the sample matrices did not affect the analyte quantitation. Thus, the proposed method was appropriate for the quantitative analysis of charantin in *M. charantia* extracts.

### 2.3. Fe_3_O_4_@MIPs and Paper@MIPs

According to a previous study [20], a combination of MIPs and interpenetrating polymer networks (IPNs) is capable of more specific binding to the analyte and efficient interference removal. This network is composed of two or more polymers that are at least partially interlaced on the molecular scale without covalent bond formation. However, the polymer network cannot be separated unless the chemical bonds in the polymer break. The synthesis procedure used the in situ radical polymerization. β-sitosterol glucoside was selected as a template owing to its larger surface area than of 5,25-stigmastedienol glucoside, which provides a sufficient binding cavity with selective recognition of all charantin components (Figure 1). Factors (i.e., types of porogenic solvents and crosslinkers, amount of template and types of substrates for MIP immobilization and amount of the immobilized MIPs) were evaluated using imprinting factors (IF) and extraction efficiency (EE).

**Figure 4:**
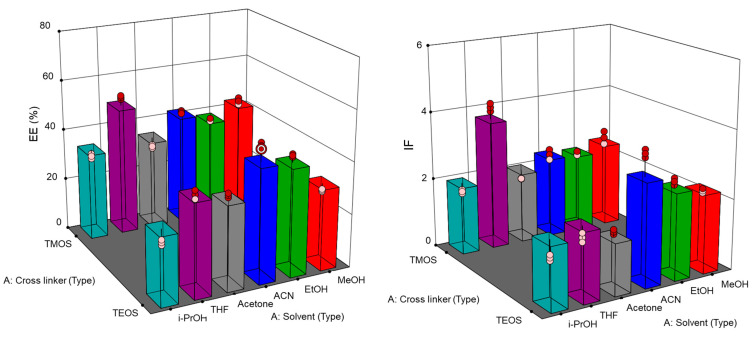
Effect porogenic solvents and cross-linkers on the imprinting factor (IF) and extraction efficiency (EE) using multilevel categoric factorial models.

Initially, Fe_3_O_4_@MIPs were synthesized at a stoichiometric ratio of 1:9:12 (template: styrene: crosslinker). The variation in the types of porogenic solvents and cross-linkers led to different effects on the performance of MIPs. The multilevel categoric factorial models of porogenic solvents and crosslinkers were significant for IF and EE with F- and *p*-values of ≥48.99 and <0.0001, respectively (Table S3). The models showed suitably adjusted R^2^ and predicted R^2^ values, indicating a good fit with the experimental data. The preparation of Fe_3_O_4_@MIPs using tetraethyl orthosilicate (TEOS) as a crosslinker resulted in the highest IF and EE values in ACN. However, tetramethyl orthosilicate (TMOS) exhibited the highest IF and EE values in tetrahydrofuran (THF), which were greater than those of the optimal Fe_3_O_4_@MIPs obtained from TEOS in ACN (Figure 4). Among the porogenic solvents, THF and ACN are aprotic solvents that can dissolve monomers, resulting in large surface areas and porous MIPs with relatively large pore sizes [29]. However, i-propanol (i-PrOH) showed the lowest extraction performance in both MIP preparation using different crosslinkers because it prevented hydrogen bond formation in the polymerization solution [30]. Hence, the preparation of Fe_3_O_4_@MIPs using TMOS in THF (EE of 53.4 ± 0.7% and IF of 4.1 ± 0.1) was utilized for further optimization.

**Figure 5:**
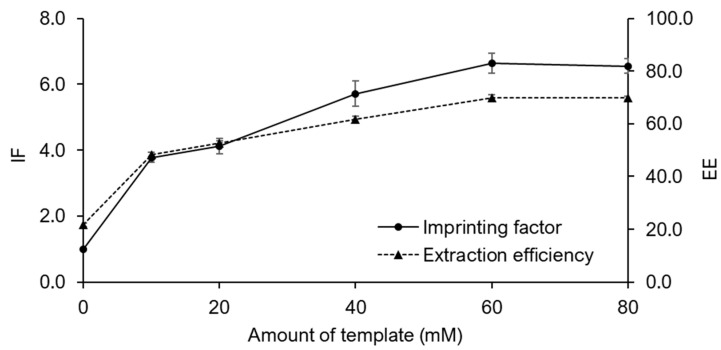
Effect of template amount on the imprinting factor (IF) and extraction efficiency (EE).

The Fe_3_O_4_@MIPs obtained by increasing the template concentration from 10 to 60 mM increased the specific binding of charantin (Figure 5). However, increasing the template concentration to 80 mM did not increase the number of specific cavities of charantin, as the EE (70.0 ± 0.9%) was not improved. Therefore, a template concentration of 60 mM was selected as the optimal concentration for the synthesis of Fe_3_O_4_@MIPs (template:styrene:crosslinker = 1:4:12). The EE was enhanced by increasing the amount of Fe_3_O_4_ magnetic nanoparticles (MNPs) used for MIP immobilization from 20 to 50 mg. The maximum EE of 87.5 ± 2.1% was achieved at 40 mg Fe_3_O_4_ MNPs indicating that the immobilized MIP yielded sufficient specific binding sites for the binding of the total target analyte. Moreover, EE did not improve with 50 mg of Fe_3_O_4_ MNPs.

Finally, paper@MIPs were prepared by the direct deposition of the reaction mixture onto a paper support to reduce the solvent evaporation time. Sequential transfer of 20 μL of the reaction mixture solution to 12 mm paper discs was performed 5 to 10 times for each paper disc. The MIP volume of 160 μL (8 × 20 μL) yielded the maximum EE of 85.0 ± 2.9%, which did not differ significantly from that of Fe_3_O_4_@MIPs. A larger amount of immobilized MIPs did not improve the EE because the paper surface area was completely accommodated with MIPs. Subsequently, the paper@MIPs were placed on a syringe filter holder connected to the syringe, which decreased the extraction time owing to accelerated analyte capture on the MIP sorbent from the pressurized syringe.

**Figure 6:**
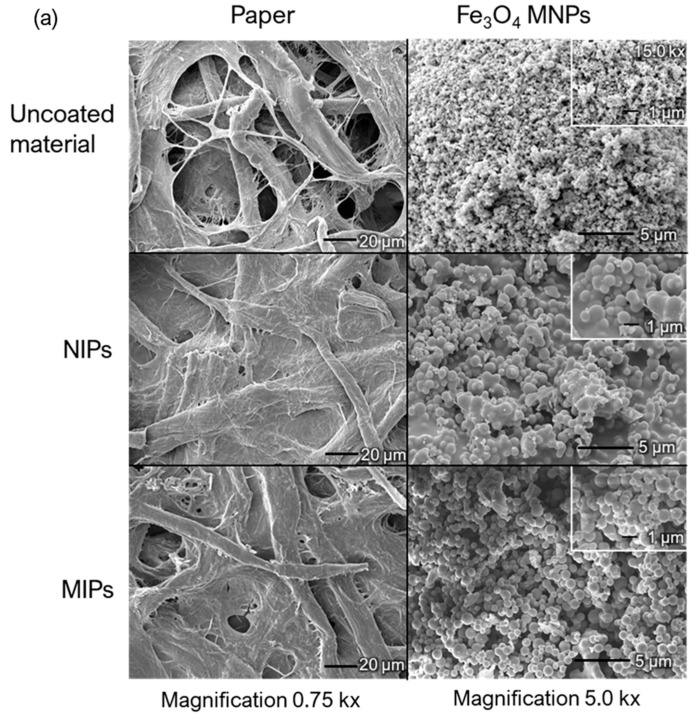

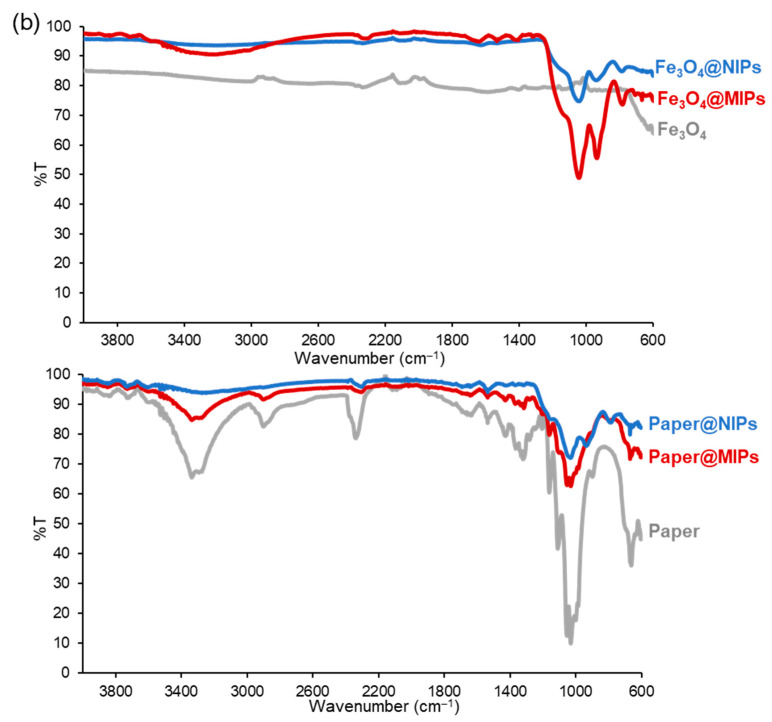
(**a**) Scanning electron microscopy images of the cellulose filter paper and Fe_3_O_4_ magnetic nanoparticles (MNPs) uncoated and coated with the non-molecularly imprinted polymers (NIPs) and molecularly imprinted polymers (MIPs) and (**b**) Fourier transform infrared spectra of the cellulose filter paper and Fe_3_O_4_ MNPs uncoated and coated with the NIPs and MIPs.

Figure 6a shows SEM images of the uncoated Fe_3_O_4_ MNPs, cellulose paper, and the same materials coated with the synthesized non-molecularly imprinted polymers (NIPs) and MIPs. The Fe_3_O_4_@NIPs structures were more rigid and denser than the Fe_3_O_4_@MIPs, indicating that the template molecules influenced the surface topography. Consequently, the Fe_3_O_4_@MIPs exhibited a uniform particle size and higher porosity, which facilitated mass transport between the solution and the surface of the Fe_3_O_4_@MIPs as SPE adsorbents. Similarly, the MIPs coating the cellulose fibers and filling the interfiber network showed more porosity than paper@NIPs. Moreover, a rigid polymer was obtained in paper@NIPs, which presumably exhibited reduced porosity and, thus, a lower affinity for the target analyte. In addition, the FTIR spectra showed different characteristics of Fe_3_O_4_ MNPs and cellulose paper and these materials were coated with the synthesized NIPs and MIPs (Figure 6b). In particular, bare cellulose paper exhibited strong broad -OH stretching at approximately 3300 cm^−1^, while decreased absorption bands were observed for paper@MIPs and paper@NIPs. The results demonstrated a reduction in free -OH groups of cellulose paper after surface modification, and paper@MIPs showed a stronger band than paper@NIPs because of their higher porosity, which enabled greater light penetration depth.

### 2.4. Applications

The newly developed method was applied for the extraction and analysis of charantin from the dried powder of *M. charantia* fruits and herbal products. Using the proposed Fe_3_O_4_@MIP-based D-µSPE, the charantin content yielded from *M. charantia* powders from two sources (obtained from P1 and P2) was higher than that obtained using the traditional LLE with *p*-values < 0.05 (Table S4). Furthermore, the methods can be reliably used for the quantitative determination of charantin in various herbal products with complex matrices. This is due to the selective binding of charantin in the complex extract using the specific recognition imprinted on the polymer, and the effective removal of potential interferences such as sterol glucoside derivatives, which are generally found in the *M. charantia* fruit extract, and other unknown compounds in the herbal product containing structurally related sterol glucosides. Additionally, the polymer matrices assist in the non-specific removal of other interferences. Therefore, the methods were effective and selective for the extraction of charantin, as well as more convenient by minimizing the steps involved in sample preparation leading to lower sample loss compared to traditional LLE. Moreover, the proposed methods reduced the time and solvent consumption.

**Figure 7:**
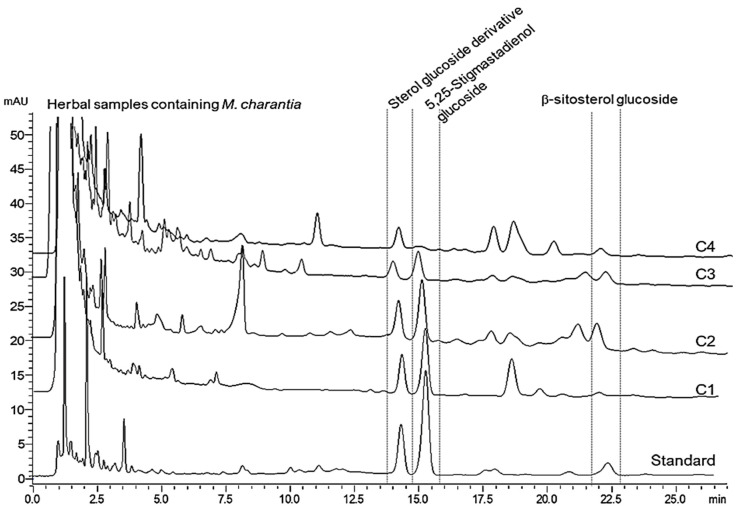
High-performance liquid chromatographic analysis of charantin in herbal products using Fe_3_O_4_@MIPs as extraction sorbents.

Figure 7 shows the herbal products containing different charantin constituents and amounts. The C1 product contained the highest amount of charantin, whereas the C4 product comprising the peptide extract from *M. charantia* seeds, contained the lowest amount [31] (Table 1). In addition, paper@MIPs were successfully applied for the extraction of charantin from the C1–C4 products and there was no statistically significant difference compared to Fe_3_O_4_@MIP-based D-µSPE in terms of extraction efficiency and recovery (*p*-values = 0.19–0.56). The methods were reproducible with %RSDs of 1.2–3.9 and 2.5–4.7 for Fe_3_O_4_@MIP-based D-µSPE and paper@MIPs, respectively, and were convenient to use compared to traditional LLE. However, the paper@MIPs offered the reduced extraction time due to the accelerated analyte capture on the MIP sorbent from the pressurized syringe. The proposed methods showed promising results and would potentially be integrated into the quality control process for the standardization of herbal products.

Notably, the results (Table 1 and Table S4) indicated that charantin is not a 1:1 mixture of stigmasterol and β-sitosterol glucosides [32]. Similarly, the previous study reported that the charantin extracts from various sources contained stigmasterol and β-sitosterol glucosides of 45.48 ± 0.28 and 110.20 ± 0.52, 68.20 ± 0.05 and 172.34 ± 0.15, 0.00 and 148.21 ± 0.71 mg/g extract [5], which were not the 1:1 mixture.

## 3. Materials and Methods

### 3.1. Chemicals

Fresh *M. charantia* fruit was obtained from Phetchaburi province, Thailand (P1). Then, seeds were removed prior to dry and grind the fruits into powder. Dried powder of *M. charantia* was purchased from TPC Herb (Bangkok, Thailand) (P2) and 3 commercial brands of capsules containing *M. charantia* powder (C1–C3) and another brand of softgel capsule (C4) containing coconut oil 165 mg, Insumate^®^ peptide extract of *M. charantia*, Sacha Inchi oil, and Gynostemma extract were from the markets in Bangkok, Thailand. ACN, acetone, THF, MeOH, ethanol (EtOH), and i-PrOH of analytical reagent (AR) and HPLC grades were purchased from Honeywell (Seoul, Republic of Korea). Hexane and hydrochloric acid (HCl) were from Supelco (St. Louis, MO, USA). Β-sitosterol, styrene, and TEOS were purchased from Tokyo Chemical Industry (Tokyo, Japan). Β-sitosterol glucoside and charantin (a mixture of 5,25-stigmastadienol β-D-glucoside and β-sitosterol β-D-glucoside) were from Chengdu Biopurify Phytochemicals Ltd. (Chengdu, Sichuan, China) and ChromaDex (Los Angeles, CA, USA). TMOS, 0.2 M 2,2′-azobis(2-methyl-propionitrile) solution (AIBN) and Fe_3_O_4_, MNPs of 20 nm and 5 µm were purchased from Sigma-Aldrich (St. Louis, MO, USA). Ethanol and sterile water for injection were purchased from Merck (Darmstadt, Germany) and General Hospital Products (Pathum Thani, Thailand), respectively.

### 3.2. Preparation of Standard Solution

All stock standard solutions of β-sitosterol glucoside and charantin were separately prepared by transferring the desired amount of standard to a 5-mL volumetric flask and adjusted to a volume with methanol. A working standard solution was prepared by diluting the stock standard solution with mobile phase for HPLC analysis. For the MIP preparation, the working standard solution of the template was diluted with the solvent used in the synthesized procedure.

### 3.3. Optimization of Fe_3_O_4_@MIPs and Paper@MIPs

Firstly, template selection was carried out by drawing three-dimensional structures of 5,25-stigmastadienol glucoside and β-sitosterol glucoside by ChemBio3D Ultra12.0, CambridegeSoft, PerkinElmer^®^ (Cambridge, MA, USA). Then, the molecular surface of two sterol compounds was evaluated, and the structure offering a larger molecular surface area was selected as a template for the synthesis of MIPs.

A hybrid of homogeneous polystyrene and silica gel polymer was synthesized by in situ radical polymerization. Firstly, a solution containing 390 μL TMOS, 762.5 µL AIBN, and 390 μL 0.1 M HCl was mixed in 4 mL ACN by stirring before the addition of 220 μL styrene monomers and template solution. The NIPs were also prepared with the absence of template [20]. Subsequently, 20 mg Fe_3_O_4_ MNPs (particle size of 20 nm) was added to the reaction mixture for the preparation of Fe_3_O_4_@MIPs/NIPs. The reaction was set at 60 °C for 4–5 h or until complete solvent evaporation, whereas 20 μL of the mixture solution was sequentially pipetted to 12 mm paper discs (5–10 times × 20 μL for each disc), which were pre-punched from Whatman^®^ filter paper No.1 sheet (GE Healthcare Ltd., Chicago, IL, USA). The complete preparation procedure of paper@MIPs/NIPs took approximately 2 h. Then, both Fe_3_O_4_@MIPs/NIPs and paper@MIPs/NIPs were washed with 10 mL each of MeOH and deionized water 4 times in order to remove the template and unreacted monomers. The HPLC analysis of the eluates was performed to ensure the complete removal (Figure S2).

Optimizations of MIPs were performed by the investigation of parameters affecting the IF and EE, i.e., types of cross-linkers (TMOS and TEOS), types of porogenic solvents (MeOH, EtOH, i-PrOH, ACN, THF, acetone), and amount of template (10–80 mM), types of substrates (Fe_3_O_4_ MNPs and paper) for MIP immobilization and the amount of coated MIPs. In addition, factorial multilevel categoric experimental design-assisted optimization of MIPs was performed to examine the effects of 2 variables including types of cross-linkers and types of porogenic solvents on the EE and IF. The statistical validity of the model was established on the basis of analysis of variance (ANOVA) by Design Expert Software^®^ 13 (Minneapolis, MN, USA). The optimal factor and level obtained from the model were further investigated. All experiments were performed in triplicate. The optimal MIPs were determined from: IF = D_MIP_/D_NIP_ = (B_MIP_/F_MIP_)/(B_NIP_/B_NIP_) = (n_bound, MIP_/n_free, MIP_)/(n_bound, NIP_/n_free, NIP_), where D_MIP/NIP_: distribution ratios, B_MIP/NIP_: concentration of bound analyte, F_MIP/NIP_: concentration of free analyte in solution, n_bound, MIP/NIP_: amount of bound analyte, n_free, MIP/NIP_: amount of free analyte in solution and EE = (extracted standard amount/spiked standard amount) × 100 [33].

The morphology of the Fe_3_O_4_@MIPs/NIPs and paper@MIPs/NIPs was observed by SEM (model JEOL JSM-6610LV, Oxford X-Max 50, Tokyo, Japan) using 10.0 kV at 750×, 5000× and 15,000× magnification. Furthermore, the characterization of Fe_3_O_4_@MIPs/NIPs and paper@MIPs/NIPs surfaces were carried out by FTIR analysis in the range of 4000–600 cm^−1^ (iS5 FTIR spectrometer with iD7 attenuated total reflection (ATR), Thermo Fisher Scientific, Waltham, MA, USA).

Moreover, the extracted charantin content from the proposed method was compared with those obtained from traditional LLE. The LLE was performed by transferring 100 mg dried powder of *M. charantia* to 2 mL hexane and mixing for 2 min. Then, the mixture was centrifuged at 5000 rpm for 5 min (Heraeus Labofuge 200, Thermo Fisher Scientific, Waltham, MA, USA) and the supernatant was removed. This step was repeated twice before the addition of 2 mL 80% MeOH. Next, the mixture was centrifuged at 5000 rpm for 5 min and the supernatant was filtered through 0.45 µm syringe filter prior to HPLC analysis.

### 3.4. HPLC-DAD Optimization

The HPLC analyses were carried out on Shimadzu HPLC system equipped with a DGU-20A5R degassing unit, Prominence LC-20AD pumping system, SIL-10AD VP autoinjector, and SPD-10A VP UV/VIS detector (Shimadzu Scientific Instruments, Kyoto, Japan). Integration and system parameters were controlled by LC solution software (Shimadzu Scientific Instruments, Kyoto, Japan). Separation was performed on an InertSustain^®^ reversed phase C-18 column with 150 mm × 4.0 mm i.d., 3 µm particle (GL Sciences, Tokyo, Japan). The injection volume was 20 µL and the detector was set at the wavelength of 204 nm. Initially, an elution was performed using isocratic solvent systems with a flow rate of 0.5 mL/min at 25 °C and the mobile phase consisted of MeOH: water (98:2, *v*/*v*). Then, the optimization was performed by varying flow rate (0.6–1.0 mL/min), oven temperature (25–50 °C), and mobile phase compositions (MeOH/water 95:5–98:2, *v/v* and ACN/water 80:20–85:15, *v*/*v*). The optimized HPLC conditions were determined from Rs, TF, number of theoretical plates (N), and relative standard deviation (RSD) of the peak area.

The unidentified peaks of charantin were characterized by LC-MS-MS analysis using the LC system (Dionex Ultimate 3000, Thermo Fisher Scientific, MA, USA) equipped with MS (Impact^TM^ II, Bruker Daltonics GmbH & Co. KG, Bremen, Germany). Separation was performed on C18 column with 100 mm × 2.1 mm, 2.6 μm particle (Dionex Acclaim^TM^ RSLC 120, Thermo Fisher Scientific, MA, USA) at 30 °C, with an injection volume of 5 μL. The mobile phases consisted of 0.1% formic acid (A) and MeOH (B). The flow rate was 0.4 mL/min and the gradient program was as follows: 80–99% B (0–10 min), 99% B (10–15 min), 99–80% B (15–15.1 min), and 80% B (15.1–20 min). The MS was performed using an ESI in positive mode as an ESI source. The ion spray voltage was set at 4500 V and the source temperature was 220 °C.

### 3.5. Method Validation

The optimized HPLC conditions were validated in terms of specificity, linearity, accuracy, precision and LOQ, according to Eurachem guide [34]. The specificity test was performed by peak purity assessment of major peaks. A calibration curve of charantin at 1–50 μg/mL was established by plotting peak areas against six different concentrations. Triplicate injections were performed for each concentration. Repeatability and intermediate precision were evaluated at the same concentration level by performing triplicate injections of each level on two different days. Method precision was presented as percent relative standard deviation (%RSD). The accuracy was reported as percent recovery (%R), which was assessed by spiking three different concentrations of charantin into the sample solutions (*n* = 3). The LOQs were based on signal-to-noise (S/N) ratio of 10.

### 3.6. Applications

The feasibility of the developed methods was demonstrated for the analysis of charantin in various samples including dried powders of *M. charantia* fruits collected from 2 sources in Thailand (defined as P1 and P2) and 4 commercial brands of herbal products (i.e., 2 brands of hard capsules contained dried powder of *M. charantia* fruits, 1 brand of hard capsule contained dried powder of *M. charantia* fruits and other unspecified herbs, and 1 brand of softgel capsules contained peptide extract from *M. charantia* seeds, coconut oil, Sacha Inchi oil, and Gynostemma extract). All experiments were performed in triplicate. Moreover, the comparison of different extraction methods (LLE vs. the developed methods) was statistically analyzed using the independent-samples t-test at the 95.0% confidence interval (SPSS Statistics 21 (IBM, Armonk, NY, USA)). The overall methodology of this work is summarized in Supplementary Figure S3.

## 4. Conclusions

Fe_3_O_4_@MIPs and paper@MIPs, which provide specific recognition sites on the polymer for target analyte binding, coupled with HPLC-DAD were successfully applied for the analysis of charantin in both the dried powder of *M. charantia* fruits and herbal products containing *M. charantia* and other medicinal plants. This method demonstrates promising prospects for environmentally friendly Fe_3_O_4_@MIPs and paper@MIPs, as they require a low amount of organic solvent for highly efficient and rapid sample preparation and analyte determination in complex matrices. Compared to previous studies, the proposed method provided more convenience by minimizing the sample preparation steps and, consequently, reducing the sample loss associated with the procedure (Table 2). In addition, Fe_3_O_4_ MNPs and paper matrices allow facile surface modification, cost effectiveness, portability, and require low storage space. Notably, with its superiority in the resolving power, the chromatographic method facilitated the separation of the three major components of charantin. However, the newly separated compound might presumably be a sterol glucoside derivative, which requires further investigation. Finally, the proposed method could be used to further develop selective µSPE and analytical methods for the quantitative analysis of other markers in herbal samples to ensure their quality.

## Figures and Tables

**Table 1:** Charantin content extracted from herbal samples by Fe_3_O_4_@MIPs and paper@MIPs.

Charantin Constituent	Content ^a^ (mg/100 g Dry Weight)							
	Fe_3_O_4_@MIPs				Paper@MIPs			
	C1	C2	C3	C4	C1	C2	C3	C4
Sterol glucoside	152.0 ± 7.1	48.9 ± 1.3	15.9 ± 2.0	24.5 ± 2.6	143.2 ± 6.4	46.8 ± 3.2	18.6 ± 2.9	23.3 ± 2.0
5,25-Stigmastadienol glucoside	365.9 ± 10.2	83.3 ± 2.6	45.4 ± 2.6	5.1 ± 0.9	357.3 ± 9.9	80.7 ± 2.9	48.1 ± 3.0	3.9 ± 0.6
*β*-sitosterol glucoside	61.0 ± 3.2	36.1 ± 0.9	197.5 ± 21.6	25.3 ± 6.2	58.7 ± 2.5	38.1 ± 2.0	183.6 ± 10.5	21.1 ± 3.5

^a^ Data are expressed as mean ± SD (*n* = 3).

**Table 2:** Comparison of the proposed method with the previous studies.

Extraction Method	Reagent Consumption ^a^	Extraction Time	Equipment Required	Analytical Method	Ref.
Fe_3_O_4_@MIPs and paper@MIPs as µSPE	10 mL methanol	10 min	Vortex, magnet separator, syringe filter holder	HPLC-DAD	The proposed method
Ultrasonic-assisted LLE	5 mL 80% methanol (46 °C)	2 h	Ultrasonicator	HPTLC densitometry	[12]
LLE	20 mL hexane, 10 mL methanol	≈1.5 h	Vortex, ultrasonicator, centrifuge	HPLC-DAD	[3]
Pressurized liquid extraction	40–60 mL ethanol (100–120 °C) or 60 mL 50% ethanol (100 °C), 5 mL 50% methanol, 5 mL 70% methanol, 3 mL hexane for purification	>40 min–1 h	Ultrasonicator, centrifuge, in-house pressurized liquid extractor	HPLC-DAD	[10]
Soxhlet extraction	200 mL ethanol, 30 mL methanol (78.5 °C), 5 mL 50% methanol, 5 mL 70% methanol, 3 mL hexane for purification	>2 h	Soxhlet apparatus, ultrasonicator, centrifuge	HPLC-DAD	[10]
Soxhlet extraction	10 mL ethanol or water (78–100 °C)	6 h	Soxhlet apparatus, ultrasonicator, centrifuge	HPLC-DAD	[11]
Shaking water bath	N/A ^b^ mL water (80 °C)	6.5 h	Shaking water bath, ultrasonicator, centrifuge	HPLC-DAD	[11]
Supercritical carbon dioxide extraction	N/A mL water and ethanol (65 °C)	≈3 h	Supercritical carbon dioxide extraction system, ultrasonicator, centrifuge	HPLC-DAD	[11]

^a^ Calculated based on 1 g of dried powder. ^b^ N/A: data not available.

## Data Availability

The data presented in this study are available on request from the corresponding author.

## Supplementary Materials

The supporting information can be downloaded at: https://www.mdpi.com/article/10.3390/ijms24097870/s1.
