# Supplementary material for: Optimization of Magnetic and Paper-Based Molecularly Imprinted Polymers for Selective Extraction of Charantin in Momordica charantia
Source: Int J Mol Sci. 2023 Apr 26;24(9):7870. doi: 10.3390/ijms24097870 (PMC10178129; doi:10.3390/ijms24097870)
Supplement: Supplementary file 1 [file ijms-24-07870-s001.zip › ijms-2305414-supplementary.pdf]

**Supplementary data**

**Table S1.** Analytical performances of HPLC analysis of 25 μg/mL charantin

| Peak no. | Name | RT (min) | Area | $R_s$ | TF | k' | N |
|---|---|---|---|---|---|---|---|
| 1 | Sterol derivative | 14.5 | 95,286 | - | 1.0 | 0 | 16,992 |
| 2 | 5, 25-Stigmastadienol glucoside | 15.5 | 227,880 | 2.0 | 0.9 | 0.065 | 16,401 |
| 3 | *β*-Sitosterol glucoside | 22.4 | 37,287 | 12.5 | 1.0 | 0.544 | 19,278 |

**Table S2**. Validation data

| Parameter | Results | |
|---|---|---|
| Calibration curve | Range (μg/mL) | *r* |
| • Sterol derivative | 1-50 | 0.9979 |
| • 5,25-Stigmastadienol glucoside | 1-50 | 0.9990 |
| • *β*-Sitosterol glucoside | 1-50 | 0.9993 |
| • Charantin | 1-50 | 0.9994 |
| Precision | %RSD | |
| • Repeatability | | |
| - Sterol derivative | 0.9-2.7 | |
| - 5,25-Stigmastadienol glucoside | 1.1-3.1 | |
| - *β*-Sitosterol glucoside | 1.0-3.5 | |
| - Charantin | 0.8-3.4 | |
| • Reproducibility | | |
| - Sterol derivative | 1.2-3.7 | |
| - 5,25-Stigmastadienol glucoside | 3.0-3.9 | |
| - *β*-Sitosterol glucoside | 2.0-3.8 | |
| - Charantin | 1.1-3.7 | |
| Accuracy | | |
| • %Recovery | Mean recoveries ± SD | |
| - Sterol derivative | 101.7 ± 2.4 to 106.1 ± 1.9 | |
| - 5,25-Stigmastadienol glucoside | 96.9 ± 2.6 to 101.9 ± 2.1 | |
| - *β*-Sitosterol glucoside | 97.7 ± 1.2 to 102.3 ± 3.0 | |
| - Charantin | 98.9 ± 2.5 to 101.9 ± 3.2 | |
| LOQs | μg/mL (%RSD) | |
| • Sterol derivative | 1.0 (2.7) | |
| • 5,25-Stigmastadienol glucoside | 1.0 (2.8) | |
| • *β*-Sitosterol glucoside | 0.5 (3.1) | |
| LODs | μg/mL | |
| • Sterol derivative | 0.34 | |
| • 5,25-Stigmastadienol glucoside | 0.34 | |
| • *β*-Sitosterol glucoside | 0.17 | |

**Table S3.** Fit statistics of the model for the measured responses

| Responses | F-value | *p*-value | $R^2$ | Adjusted $R^2$ | Predicted $R^2$ | Adeq. Precision |
|---|---|---|---|---|---|---|
| Imprinting factor (IF) | 48.99 | < 0.0001 | 0.9169 | 0.8982 | 0.8676 | 26.2467 |
| Extraction efficiency (EE) | 103.27 | < 0.0001 | 0.9588 | 0.9495 | 0.9381 | 38.0388 |

**Table S4.** Charantin content extracted from *M. charantia* powder by liquid-liquid extraction (LLE) and $Fe_3O_4$@MIPs

| Charantin constituent | Content[a] (mg/100 g dry weight) | | | | | |
|---|---|---|---|---|---|---|
| | P1 | | *p*-value | P2 | | *p*-value |
| | LLE | $Fe_3O_4$@MIPs | | LLE | $Fe_3O_4$@MIPs | |
| Sterol glucoside | 53.0 ± 9.8 | 135.2 ± 7.1 | 0.000 | 44.2 ± 2.4 | 112.4 ± 15.6 | 0.002 |
| 5,25-Stigmastadienol glucoside | 51.3 ± 7.6 | 126.0 ± 5.8 | 0.000 | 45.9 ± 2.5 | 109.1 ± 14.1 | 0.002 |
| *β*-Sitosterol glucoside | 18.7 ± 3.3 | 38.5 ± 2.3 | 0.001 | 123.4 ± 4.7 | 179.5 ± 14.9 | 0.003 |

[a] Data are expressed as mean ± SD (n = 3).

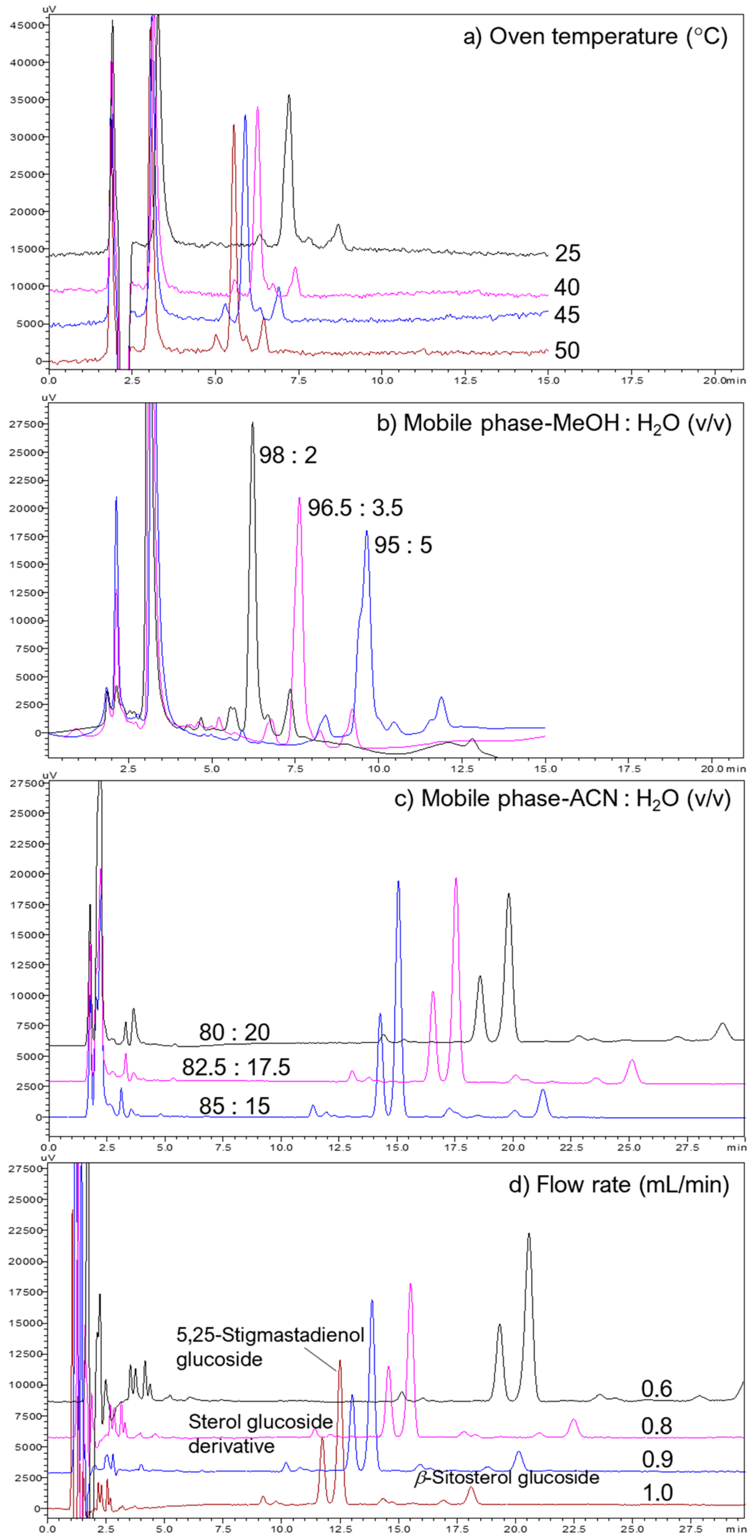


**Figure S1.** Effects of **(a)** oven temperature, **(b)** methanol content in mobile phase, **(c)** acetonitrile content in mobile phase and **(d)** flow rate on the chromatographic separation of charantin.

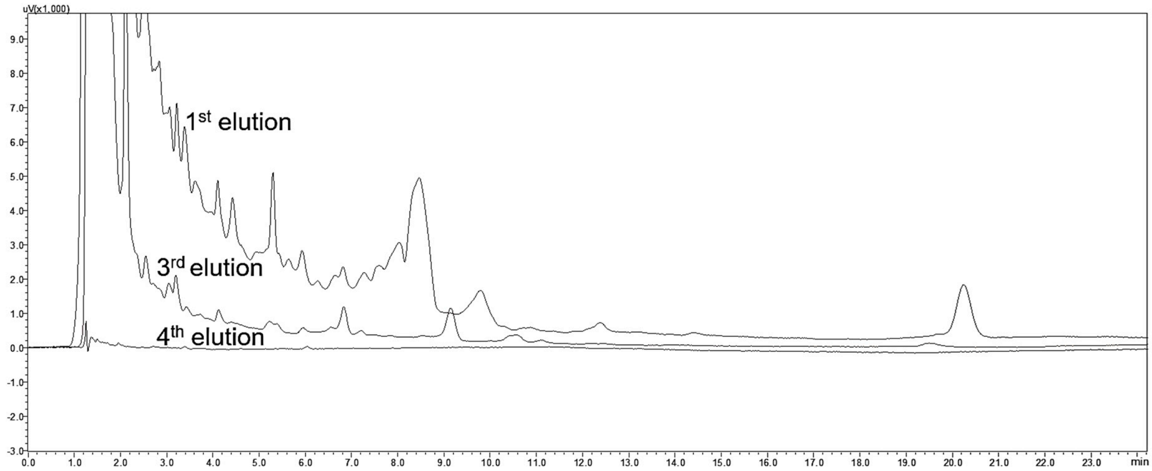


**Figure S2.** HPLC analysis of eluates obtained from the washing step of MIPs for removal of the template and unreacted monomers.

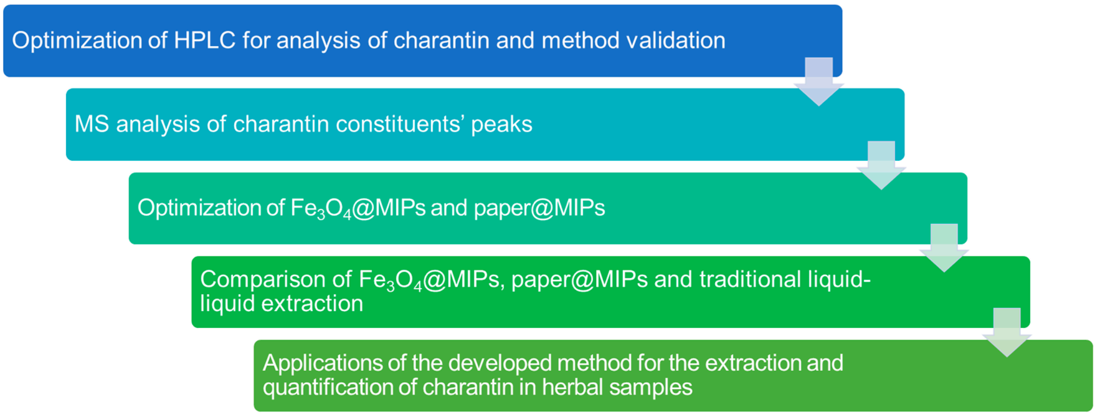


**Figure S3**. Overall methodologies
